# Artificial intelligence in cardiovascular medicine: An updated review of the literature

**DOI:** 10.34172/jcvtr.2023.33031

**Published:** 2023-12-30

**Authors:** Arian Zargarzadeh, Elnaz Javanshir, Alireza Ghaffari, Erfan Mosharkesh, Babak Anari

**Affiliations:** ^1^Central Toronto Academy, Toronto, Canada; ^2^Cardiovascular Research Center, Tabriz University of Medical Sciences, Tabriz, Iran; ^3^Faculty of Electrical and Computer Engineering, University of Tabriz, Tabriz, Iran; ^4^Faculty of Veterinary Medicine, University of Tabriz, Tabriz, Iran; ^5^Department of Computer Engineering, Shabestar Branch, Islamic Azad University, Shabestar, Iran

**Keywords:** Artificial intelligence, Cardiovascular disease, Healthcare

## Abstract

Screening and early detection of cardiovascular disease (CVD) are crucial for managing progress and preventing related morbidity. In recent years, several studies have reported the important role of Artificial intelligence (AI) technology and its integration into various medical sectors. AI applications are able to deal with the massive amounts of data (medical records, ultrasounds, medications, and experimental results) generated in medicine and identify novel details that would otherwise be forgotten in the mass of healthcare data sets. Nowadays, AI algorithms are currently used to improve diagnosis of some CVDs including heart failure, atrial fibrillation, hypertrophic cardiomyopathy and pulmonary hypertension. This review summarized some AI concepts, critical execution requirements, obstacles, and new applications for CVDs.

## Introduction

 Artificial Intelligence (AI) refers to the progress of computer science that can think or act like humans and administers functions that usually require human intelligence, including perception, cognitive, reasoning, and controlling.^1^ Actually, AI can mimic the human brain for processing and operating data and start to perform an impressive role in medicine and health with facilitating in identification, processing, integration, and analyzing of various amounts of healthcare data.^2^

 Medical imaging and diagnostics, rehabilitation, medical research and drug discovery, patient engagement and compliance are the various applications of AI in the field of healthcare.^3^ In addition, AI has been used for providing personalized health information, enabling virtual consultations and remote monitoring and management.^4^ Machine learning (ML), artificial neural networks, convolutional neural networks (CNNs), cognitive computing, and deep learning are main subdisciplines of AI. ML, a more prominent subset of AI as a whole, is the process of creating algorithms and models that can be trained on massive datasets in order to find patterns, forecast results, and diagnose conditions.^5^ Generally, ML enhances the precision of medical professionals’ diagnosis and aids in their decision-making.^6^

## Artificial intelligence in cardiovascular diseases

 AI, defined as the application of advanced computer algorithms to extract information from complicated datasets, is currently in use in a variety of medical sectors, including research, diagnosis and therapy.^7^ As with other disorders like diabetes and cancer, the prevalence of cardiovascular disease (CVDs) is rising in today’s society, and it remains to be a leading cause of mortality.^8,9^ Based on pioneering reports, clinicians can use the results of specific AI-processed algorithms of electrocardiogram (ECGs) on current big data to improve diagnosis of pulmonary hypertension,^10^ heart failure,^11,12^ atrial fibrillation,^13^ and hypertrophic cardiomyopathy.^14^

**Figure 1:**
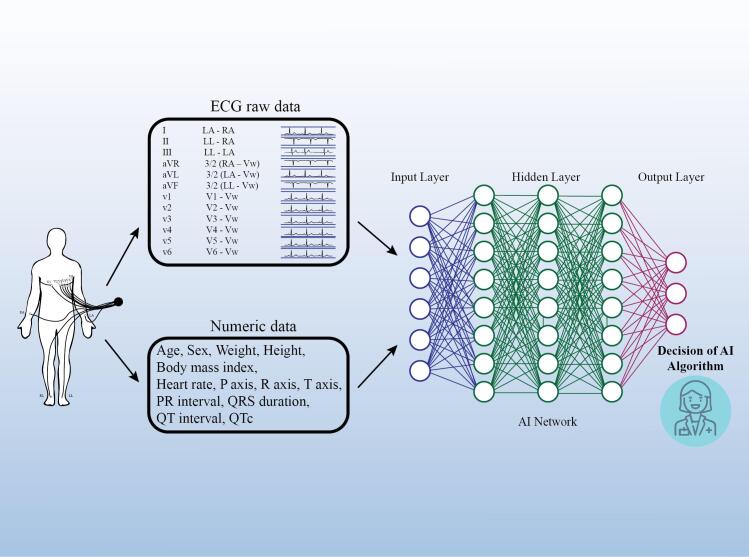
Description of artificial intelligence algorithm for detecting of cardiovascular disease. AI, artificial intelligence; ECG, electrocardiography

 In order to give physicians a better grasp and use of AI, this review attempts to condense the application of AI in CVDs from the viewpoint of the clinician. Figure 1 shows AI algorithm for detecting CVDs.

## Materials and Methods

 As suitable for narrative reviews, a literature search was conducted using electronic resources from PubMed, Scopus, and Google Scholar. We aimed to summarize the published literature on the AI in the treatment of CVDs. A combination of the terms “Artificial Intelligence,” “cardiovascular disease,” “heart disease,” “machine learning,” “hypertrophic cardiomyopathy,” “hypertension,” “coronary artery disease,” “primary pulmonary hypertension,” “heart failure,” “atrial fibrillation,” and “anemia,” was used. Exclusion criteria considered as case reports, editorials letters, and papers for which full texts were not available in English. The last of these searches was carried out on August 30, 2023.

## AI in Pulmonary Hypertension

 A varied collection of disorders known as pulmonary hypertension (PH) is characterized by a mean pulmonary artery pressure of at least 25 mm Hg at rest.^15^ The non-specific symptoms of PH are mostly associated with the increasing malfunction of the right ventricle (RV). Shortness of breath during exercise is usually the initial symptoms of PH, which follows by exhaustion, weakness, angina, and syncope.^16^ PH may have a quickly progressive clinical course with reduced exercise tolerance and dyspnea linked to right-sided heart dilatation, RV hypertrophy, and eventually, heart failure.^17^

 Echocardiography, chest radiography, and electrocardiography (ECG) are frequently used in the detection of PH. Chest radiographs are non-specific for differential diagnosis with other heart diseases, and although most patients with PH have abnormalities on their initial ECG that correspond to right heart overload, the standard ECG is insufficient for screening. Cardiac magnetic resonance imaging (CMR) is one of the selected procedures for patients under investigation for PH. It offers a precise evaluation of cardiac functional status; however, in order to fully utilize cardiac imaging’s predictive potential, techniques for identifying the most significant and pertinent prognostic features must be developed. In terms of PH screening, echocardiography is considered as a gold standard.^18^ Global recommendations suggested echocardiographic parameters when PH is suspected; nonetheless echocardiography provided valuable information in the realm of PH but these data are insufficient to confirm a diagnosis of PH.^19^ Since, PH is commonly asymptomatic or has unclear symptoms, it is critical to diagnose the condition as soon as possible to stop the disease’s irreversible progression and death.^16^ Recently, the combination of machine learning and computational image analysis may make it possible to identify the intricate functional modifications that serve as a predictor of impending right-sided heart failure and mortality.^20^ Based on recent studies the utilization of cardiac MRI for cardiac image segmentation, along with the quantification of ventricular volume, wall thickness, ejection fraction, strain imaging, and AI-assisted data analysis, has created new opportunities for the diagnosis, treatment, and risk assessment of conditions like PH.^11,21^ Evidences showed that in comparison to conventional parameters, a machine-learning survival model that takes heart motion into account has increased prognostic potential. Based on controlled machine learning of cardiac motion patterns, a reduction of effective contractile motion in anatomically separate but functionally linked areas of the right ventricle predicts survival in PH patients.^20^ Clinical data sets can be used for both supervised and unsupervised machine learning techniques to create reliable risk models and redefining patient classes.^22^ PH conventional cardiac machine learning studies have demonstrated that machine-learning algorithm and an advanced 3D model of cardiac displacement can find recurrent patterns in a high-dimensional data set more efficient in predicting the outcomes.

 Kwon et al demonstrated based on a sensitivity map that in patients with a narrow QRS deep learning-based AI algorithm concentrated on the QRS complex, particularly the S-wave can have a strong correlation with PH. In other words, their findings showed that in comparison to patients without PH, those with PH had greater QRS durations and right axis deviation.^10^ Furthermore, other findings showed that PH could be detected and predicted with a single-lead (especially based on the right precordial leads (V2−V4)) wearable device that uses the AI algorithm, as well as with a conventional 12-lead ECG.^10,20^ Considerable data from recent studies suggests that the AI algorithm could predict the screen of PH in suspected patients using a simple wearable device, such as a watch or patch which can be usefulness especially in developing countries with limited medical resources.

## AI in Atrial Fibrillation

 Atrial fibrillation (AF), especially paroxysmal AF is a common and morbid arrhythmia, with an increased risk of unfavorable outcomes including heart failure, stroke, dementia, and some other cardiovascular diseases.^23^ Due to the asymptomatic and elusive nature of the disease, when an ECG is recorded, patients with AF frequently exhibit a normal sinus rhythm, which may result in a misdiagnosis. Furthermore, current screening techniques are expensive, yield-limited, and need constant observation.^24^ Nevertheless, according to recent studies, a deeply trained neural network may be able to detect subtle changes in the normal sinus-rhythm of ECGs in order to predict AF ^13^. Previous observations claimed that AI-ECG is a helpful tool for determining the likelihood of developing AF. Interestingly, through risk factor stratification assessment, AI can also identify future AF.^25,26^ In a study by Khurshid et al.^25^ it has been showed that a CNN called ECG-AI is a useful tool for forecast 5-year AF-free survival. In that study, a single 12-lead ECG with a time-series of 5,000 voltage measurements for each of the 12 leads, sampled at 500 Hz and lasting 10 seconds, serves as the input for this ECG-AI. In addition, their results generally support the idea that deep learning models using 12-lead ECG provide new evidences that risk estimates are generalizable and retain predictive value for up to five years following an ECG performance. In summary, based on previous data when comparing the 5-year AF risk discrimination with the 11- component Cohorts for Heart and Aging Research in Genomic Epidemiology (CHARGE)–AF risk score, ECG-AI performs comparably.

## AI in Coronary Artery Disease

 Globally, coronary artery disease (CAD) continues to be the main cause of cardiovascular disease-related mortality and long-term disability. Therefore, it is critically necessary to have accurate, useful, and affordable CAD screening tools.^27^ Clinical studies showed that certain facial features were linked to a higher risk of CAD, suggesting an opportunity for disease screening. Scientific evidences showed that some factors probably are associated with increased risk of CAD and poor cardiovascular health such as alopecia, facial wrinkle, earlobe crease, grey hair, xanthelasmata, and arcus corneae.^28-30^ So, it is therefore necessary to have a tool for disease screening that incorporates all of the facial features linked to CAD. As artificial intelligence has progressed, the deep learning algorithm has emerged as a promising tool for disease diagnosis and prediction based on facial photos particularly for genetic and endocrine diseases.^31^ A deep learning algorithm based on images of human faces for CAD screening was shown to be feasible by Lin et al^27^ Furthermore, recent results claimed that the algorithm of AI could be created as a self-reported smartphone application to measure CAD risk prior to a doctor’s appointment in high-risk community populations. The findings could then be utilized to facilitate a patient-centered conversation about cardiovascular health.^32^ In a study, Betancur et al^33^ attempted to train a deep learning model from SPECT myocardial perfusion imaging (MPI) to predict future coronary artery disease (CAD). A stratified tenfold cross-validation approach was used to test the model. The results indicating that deep learning can aid in MPI analysis and prediction of future CAD.

 When compared to existing conventional approaches, deep learning improves automated prediction of obstructive CAD. To assess the practicality of AI clinical applications, further clinical and imaging data should be included in future research.

## AI in Heart failure

 Asymptomatic left ventricular systolic dysfunction (ALVSD) is associated with a 1.6-fold increased risk of all-cause mortality and a 6.5-fold increased risk of clinical heart failure (HF). It affects 1.4–2.2% of the general population.^34,35^ Echocardiography is frequently used to measure left ventricular ejection fraction (LVEF), a crucial indicator of left ventricular systolic function. It has been shown that effective treatment is crucial for improving left ventricular systolic functions, prevent further loss of LVEF and irreversible myocardial damage, as well as raise in survival and quality of life for patients with HF.^36^ Today, AI-ECG has been indicated in a number of recent studies as a useful aspect for left ventricular dysfunction screening. As known, quantitation of LVEF using 2-dimensional speckle-tracking echocardiography (2D-STE) is unsuitable due to the fact that LVEF is typically determined manually by drawing boundaries or by the traditional “eyeball” method, both of which have poor precision and reproducibility. This gap may be improved via AI with better clinical decisions.^37^ Recently investigators claimed that AI algorithm can facilitate early diagnosis of patients with low EF (it has been investigated in different studies as EF ≤ 35% or EF ≤ 50%) based on their ECG.^11,12^ In summary, more evidences guarantee the AI-ECG usefulness in screening of left or right ventricular dysfunction.

## AI in Cardiomyopathy

 One of the main causes of sudden cardiac death in young adults is hypertrophic cardiomyopathy (HCM). The risk estimates provided by current risk algorithms are imprecise and do not take into consideration the varying impact sizes of distinct risk variables.^38^ Despite the fact that echocardiography is the gold standard for the diagnosis and preliminary assessment of HCM, it is unclear how best to identify HCM in those who are asymptomatic. However, over 90% of individuals diagnosed with HCM have electrocardiographic abnormalities. Twelve-lead electrocardiography (ECG) is a viable noninvasive, affordable, and quick method of screening for the condition.^14^

 Recently, AI approaches has been considered for high diagnostic performance of HCM particularly in younger patients. AI network in large datasets, and with nonlinear models can see features that are not obvious to even an expert ECG interpreter.^11,14^ Ko et al^14^ tested an AI-based CNN approach for detection of HCM based on the 12-lead ECG. They used Python (Python Software Foundation, Beaverton, Oregon) and the Keras Framework with a TensorFlow backend (Google, Mountain View, California) to apply a convolutional neural network (CNN). They concluded that a deep learning method that uses ECG data may reliably identify individuals with HCM across a variety of subgroups, including those with normal ECG or left ventricular hypertrophy patterns particularly in patients who are younger. Consistent with prior finding, a CNN model was developed by Shrivastava et al. to enable the early identification of dilated cardiomyopathy using ECG in a population of 16,025 participants with normal LVEF and 421 patients with dilated cardiomyopathy.^39^ In summary, Electrocardiogram abnormalities are present in over 90% of HCM patients; nevertheless, they are non-specific and cannot be differentiated from other medical conditions. AI-ECG might be a useful tool for HCM diagnosis.

## Discussion

 Cardiovascular disorders are the leading cause of death and morbidity worldwide, comprising a wide range of disorders affecting the heart and its arteries.^40^ Since most CVD patients are asymptomatic or have unclear symptoms, it is critical to develop a trustworthy screening tool to identify the condition early on in order to prevent irreversible disease progression and death. At present, medicine has been altered by AI applications. AI techniques in cardiovascular medicine have shown a promising effect since it is claimed that has the power to change patient outcomes and the manner that treatment is provided.

 Compared to many other disciplines, cardiologists often have access to more quantitative patient data for making choices about patient treatment.

 Strengthening AI algorithms will support physicians discreetly and streamline clinical treatment as companion medical assistants. In addition, AI will enable clinicians to evaluate more data with greater accuracy than ever before, which will lead to better patient care.^41^ This review has indicated some distinct topics for more investigation into novel and innovative approaches to human-AI intelligent caring.

## Conclusion

 In conclusion, it is important to remember that AI in cardiovascular medicine remains in its early stages and that there are still certain issues that need to be resolved. Ensuring the accuracy and dependability of AI systems is a major concern as mistakes or errors in AI-based decision-making can have serious negative effects on patients. Furthermore, data privacy and security breaches are possible risks associated with AI in healthcare. Totally, AI has enormous potential in cardiovascular medicine, but its potential therapeutic benefit may be overwhelmed if its limitations are not recognized.

## Competing Interests

 The authors declare no conflict of interest.

## Funding

 This research received no external funding.
